# Stability of APOBEC3F in the Presence of the APOBEC3 Antagonist HIV-1 Vif Increases at the Expense of Co-Expressed APOBEC3H Haplotype I

**DOI:** 10.3390/v15020463

**Published:** 2023-02-07

**Authors:** Maria Yousefi, Arun Kumar Annan Sudarsan, Amit Gaba, Linda Chelico

**Affiliations:** Department of Biochemistry, Microbiology, and Immunology, College of Medicine, University of Saskatchewan, Saskatoon, SK S7N 5E5, Canada

**Keywords:** restriction factor, HIV-1, APOBEC3

## Abstract

The seven human APOBEC3 enzymes (APOBEC3A through H, excluding E) are host restriction factors. Most of the APOBEC3 enzymes can restrict HIV-1 replication with different efficiencies. The HIV-1 Vif protein combats APOBEC3-mediated restriction by inducing ubiquitination and degradation in the proteasome. APOBEC3F and APOBEC3G can hetero-oligomerize, which increases their restriction capacity and resistance to Vif. Here we determined if APOBEC3C, APOBEC3F, or APOBEC3G could hetero-oligomerize with APOBEC3H haplotype I. APOBEC3H haplotype I has a short half-life in cells due to ubiquitination and degradation by host proteins, but is also resistant to Vif. We hypothesized that hetero-oligomerization with APOBEC3H haplotype I may result in less Vif-mediated degradation of the interacting APOBEC3 and stabilize APOBEC3H haplotype I, resulting in more efficient HIV-1 restriction. Although we found that all three APOBEC3s could interact with APOBEC3H haplotype I, only APOBEC3F affected APOBEC3H haplotype I by surprisingly accelerating its proteasomal degradation. However, this increased APOBEC3F levels in cells and virions in the absence or presence of Vif and enabled APOBEC3F-mediated restriction of HIV-1 in the presence of Vif. Altogether, the data suggest that APOBEC3 enzymes can co-regulate each other at the protein level and that they cooperate to ensure HIV-1 inactivation rather than evolution.

## 1. Introduction

Human APOBEC3 (A3) enzymes are single-stranded (ss) DNA and RNA cytosine deaminases that act as host restriction factors with crucial roles in intrinsic immunity against certain viruses. While A3 enzymes have been primarily characterized for restricting retroviruses, such as HIV-1, they can also restrict double-stranded (ds) DNA viruses, such as Epstein-Barr virus, endogenous retroviruses, retroelements and possibly RNA viruses, such as coronaviruses [1,2,3,4,5]. The deamination of cytosine to form uracil in transiently available single-stranded (ss)DNA during virus or retroelement replication or RNA results in mutations (RNA and ssDNA) or degradation of the ssDNA [6,7]. The human A3 enzyme family has seven members named A3A, A3B, A3C, A3D, A3F, A3G, and A3H [8,9]. From these seven members, five (A3G, A3F, A3H, A3D, and A3C S188I) are able to restrict the retrovirus HIV-1 (referred to as HIV) and they have been shown to be co-expressed in CD_4_^+^ T cells [10,11,12,13].

HIV restriction ability of A3s is a combination of deamination-dependent and deamination-independent mechanisms [7,14]. The five A3 enzymes that can restrict HIV are localized to the cytoplasm, which enables them to bind HIV genomic RNA and encapsidate into newly forming HIV virions [1,6]. In the next cell these HIV virions infect, A3 enzymes deaminate cytosine to uracil on minus-sense ssDNA during reverse transcription of the RNA genome [6,15]. When HIV reverse transcriptase synthesizes the plus-strand DNA, the uracils are used as template for insertion of adenines causing guanine (G) to adenine (A) mutations in the plus-sense coding strand [6,15]. The resulting mutated pro-viral DNA can be integrated into the host genome but may be functionally inactivated [6,15]. At the same time, host DNA repair systems can remove these uracils creating basic sites on DNA, which may lead to pro-viral DNA degradation [7]. In addition, A3 enzymes can act in a deamination-independent manner by directly binding to RNA or the reverse transcriptase and delaying the initiation of primer extension or physically blocking DNA elongation and scanning of reverse transcriptase [7,16,17].

To carry out these viral restriction functions, A3 enzymes must first avoid the HIV Viral Infectivity Factor (Vif). Vif hijacks the Cullin-ring E3 ubiquitin ligase complex of the host cell and acts as the substrate receptor for A3 enzymes [1,18]. Vif also interacts with the co-transcription factor CBFβ (Core-Binding Factor subunit beta) for stability and interacts with Elongin C (Elo C) that forms a hetero-oligomer with Elongin B (Elo B) and Cullin 5 (Cul 5) [18,19,20,21]. Cul 5 interacts with RING box protein 2 (Rbx2), which recruits the E2 ubiquitin conjugating enzyme [18]. This complex leads to A3 polyubiquitination and proteasomal degradation to enhance HIV viral infectivity [1,18]. Despite the action of Vif, HIV pro-viral genomes accumulate numerous G to A mutations within the first 6 weeks of infection [22]. These data suggested that there has to be a more active mechanism than just “avoiding” Vif for such mutations to accumulate. Vif expression is highest in cells as viral packaging is occurring, suggesting that stochastic avoidance of Vif would not be efficient [23].

Previously, our lab identified that hetero-oligomerization of A3G and A3F, which can both restrict HIV independently, resulted in greater restriction of HIV and protection of A3F from Vif-mediated degradation [24,25]. The A3G-mediated protection of A3F from Vif resulted in more A3F-mediated mutations in HIV genomes in the presence of Vif [25]. It is known that the expression of A3C, A3D, A3F, A3G and A3H in CD_4_^+^ T cells is constitutive and therefore would result in multiple A3s being present at the same time, not only A3G and A3F [10,11]. Here we investigated if the other A3 hetero-oligomers form and if they affect Vif-mediated degradation of the interacting A3s. We were specifically interested in A3H.

A3H is a unique member in the A3 family. A3H forms an obligate dimer of two A3H molecules with double-stranded RNA mediating the dimer interface [26,27,28,29]. The two proteins in the dimer do not contact each other. In the absence of the RNA, A3H becomes unstable [30]. A3H also has numerous polymorphisms that has resulted in seven major haplotypes (Hap I-VII) and four splice variants (SV154/182/183/200) with variable antivirus capability [31]. A3H Hap I, the most common haplotype in humans, has been shown to be less stable in cells than other haplotypes II, V, and VII due to ubiquitination and degradation [32,33]. This degradation, and that A3H Hap I is partially localized to the nucleus, results in modest HIV restriction (approximately two-fold) [32,33,34]. In contrast, more stable haplotypes (II, V, and VII) are more localized to the cytoplasm and can strongly restrict HIV replication [33,35,36]. There are also very unstable haplotypes (III, IV, and VI) that have no functional ability and cannot be detected in cells by immunoblotting [33,35]. Interestingly, A3H Hap I is resistant to Vif-mediated degradation [31]. The full potential of this resistance is not observed since A3H Hap I is less stable [33]. We hypothesized that interaction with other more stable A3 enzymes, may enhance A3H Hap I activity. We specifically tested A3F, A3G, and A3C. While A3F and A3G are known to efficiently restrict HIV in the absence of Vif, they are also sensitive to Vif [25,37,38]. A3C does not restrict HIV, but it is highly abundant in CD_4_+ T cells [10,13].

Using a combination of molecular and virological techniques, we show that A3H Hap I can interact with A3F, A3G and A3C, but there is only a phenotype for this interaction with A3F. In contrast to A3F stabilizing A3H Hap I, we find the opposite—that the presence of A3F further destabilizes A3H Hap I. Nonetheless, the interaction still results in higher A3F levels in cells in the presence and absence of Vif. This results in two-fold more restriction of HIV by A3F both in the absence and presence of Vif. Although a two-fold change is minor in the absence of Vif, the co-expression of A3F and A3H Hap I enables restriction of HIV by two-fold in the presence of Vif by the remaining A3F whereas A3F alone has no HIV restriction capacity in these conditions. These data reveal additional complexities of HIV restriction by the A3 family and support the idea that A3 enzymes function together rather than independently to ensure HIV inactivation by becoming partially resistant to Vif-mediated degradation.

## 2. Materials and Methods

### 2.1. Plasmid Constructs

To express two A3 transcripts on a single-cell basis, we used an expression plasmid, pVIVO2 (Invivogen, San Diego, CA, USA), with two transcription units in a single vector. Constructs contained one HA-tagged A3 or one V5-tagged A3 or both. All V5-tagged A3s (A3F 108A/231V, A3F 108S/231I, A3C, and A3G) were cloned using an XbaI site in MCS1. The pVIVO2 A3F 108S/231I-V5 was previously described [24]. The cDNA for A3F 108A/231V, A3C and A3G were amplified from pcDNA constructs and a previously established cloning strategy was used to obtain V5-tagged versions of these cDNA in pVIVO2 [24,36,37,39]. The following reagent was obtained through the NIH HIV Reagent Program, Division of AIDS, NIAID, NIH: Plasmid pcDNA3.1-APOBEC3G-HA Expressing Human APOBEC3G with C-Terminal Triple HA Tag, ARP-9952, contributed by Dr. Warner C. Greene. The A3H Hap I was subcloned from a pcDNA construct into the MCS2 of pVIVO2 using NheI [40].

### 2.2. Co-Immunoprecipitation

HEK 293T cells (2.5 × 10^6^ per 75 cm^2^ flask) were transfected with 2 µg of DNA. The transfections consisted of 1µg of pVIVO2 A3H Hap I-HA expression plasmid and 1µg of A3F 108S/231I-V5, A3F 108A/231V-V5, A3G-V5 or A3C-V5 expression plasmid. GeneJuice (Novagen/EMD Millipore, Burlington, MA, USA) transfection reagent was used according to the manufacture’s instructions. After 40 h of transfection, cells were washed with cold PBS, lysed with cold immunoprecipitation buffer (50 mM Tris-Cl [pH 7.4], 1% Nonident-P40, 0.1% sodium deoxycholate, 150 mM NaCl, 10% glycerol, EDTA-free protease inhibitor (Roche, Basel, Switzerland)) and clarified by centrifugation at 4 °C. Clarified supernatants were treated with RNase A (0.1 mg/mL, Roche) at 4 °C for 90 min and the experimental condition was incubated with lysate and polyclonal anti-HA antibody (1:1000, Sigma, St. Louis, MO, USA) and the mock had no anti-HA antibody. The co-immunoprecipitation (co-IP) was washed with immunoprecipitation buffer three times before resuspending in 2× Laemmli buffer and then resolving on SDS-PAGE and transferring to nitrocellulose for immunoblotting. The blot then was probed with anti-V5 mouse antibody (1:5000, Sigma) for detecting HA-immunoprecipitated lysates. For detecting the expression of A3s in cell lysate, the nitrocellulose membrane was probed with anti-HA rabbit (1:2000, Sigma), anti-V5 mouse (1:5000, Sigma) and α-tubulin rabbit (1:2000, Sigma).

### 2.3. Single Cycle Infectivity Assay

The single-cycle infectivity assay was conducted following an established method [41]. Specifically, 1 × 10^5^ HEK 293T cells per well of a 12-well plate were co-transfected with 500 ng of HIV-1_LAI_ ΔEnv ΔVif (referred to as −Vif) or HIV-1_LAI_ ΔEnv (referred to as +Vif), 180 ng of VSV-G, and 0, 25, 50, or 100 ng of pVIVO2 plasmid (empty or A3 expressing). GeneJuice (Novagen/EMD Millipore) transfection reagent was used according to the manufacture’s protocol. Forty-eight hours post-transfection virus containing supernatants was harvested and filtered through a 0.45 µm polyvinylidene difluoride (PVDF) syringe filter. Filtered virus was used for both infection of TZM-bl (HeLa CD_4_ + CCR_5_ + LRT *lac*Z cells) reporter cells and concentration and lysis for use in immunoblots. Virus producing cells were collected for use in immunoblots. To measure the infectivity, 1 × 10^4^ TZM-bl cells per well of a 96-well plate were infected with a dilution series of filtered virus in presence of 8 µg/mL of polybrene. Then, 48 h post-infection, the infectivity was measured through colorimetric detection using β-galactosidase assay reagent (Pierce) via spectrophotometer. Infectivity of each virus was compared to the No A3 condition that was set to 100%

### 2.4. Integration of Pro-Viral DNA

Integrated pro-viral DNA was determined as previously described [41,42,43]. In brief, infections used 1 × 10^5^ HEK 293T cells per well of a 12-well plate were infected by spinoculation (1 h at 800× *g*) and in the presence of polybrene (8 μg/mL) with HIV produced from the single-cycle replication assays. DNA was extracted after 24 h using DNazol according to manufacturer’s instructions (Invitrogen, Waltham, MA, USA). The DNA was then treated with DpnI and integrated pro-viral DNA was amplified using an HIV-specific primer with Alu-specific primers to ensure episomal DNA was not amplifed [41,43]. This PCR product was diluted and used for qPCR to determine the amount of integrated pro-viral DNA [41,43].

### 2.5. Pro-Viral DNA Sequencing

For pro-viral sequencing, 1 × 10^5^ HEK293T cells per well of a 24-well plate were infected with supernatant containing virus in the presence of 8 μg/mL polybrene. The plates were spinoculated (1 h at 800× *g*). DNA was extracted after 48 h using DNazol according to manufacturer’s instructions (Invitrogen). The PCR amplification of a *polymerase* (*pol*) region of HIV (581 bp) and treatment of DNA with DpnI was carried out as previously described [41,44]. Sequences were analyzed with Clustal Omega [45] and Hypermut [46].

### 2.6. MG 132 Treatment

HEK 293T cells (1 × 10^5^) were seeded per well of a 12-well plate. After 24 h, cells were transfected with 100 ng of A3F 108A/231V-V5, A3H Hap I, A3F 108A/231V/A3H Hap I, or empty pVIVO2 plasmid. As a control, we also transfected 100 ng of A3F 108A/231V and 100 ng of pcDNA containing Vif from HIV_LAI_. Sixteen hours post-transfection the media was changed. Twenty-eight hours post-transfection, cells were treated with DMSO (Fisher Scientific, Waltham, MA, USA) or MG132 (Cayman Chemical Company, Ann Arbor, MI, USA) dissolved in DMSO (12.5 µM). Sixteen hours post-treatment, cells were harvested in 2× Laemmli buffer and analyzed by immunoblotting.

### 2.7. Immunoblotting

Immunoblotting was conducted to measure the A3 encapsidation in virions and steady state protein levels in cell lysates. Rabbit anti-HA (1:5000, Sigma) and Mouse anti-V5 (1:2000, Sigma) were used for detection of HA-tagged A3H Hap I and V5-tagged A3F 108A/231V, A3F 108S/231I, A3G, and A3C, respectively. Rabbit anti-α-tubulin (1:5000, Sigma) and Mouse anti-p24 (HIV-1 capsid protein) (1:1000, Cat#ARP-3537, NIH AIDS Reagent Program) were used as the loading controls for cell lysates and viral lysates, respectively. Secondary detection was performed using Licor IRDye antibodies produced in goat (IRDye 680 labeled anti-Rabbit, and IRDye 800 labeled anti-Mouse). Blots were scanned via LICOR CLx. For quantification, Image Studio was used to detect the pixel intensity of the experimental and loading control bands. Each sample was normalized to its own loading control before comparison to other lanes.

## 3. Results

### 3.1. A3H Hap I Interacts with Multiple A3s

**Figure 1:**
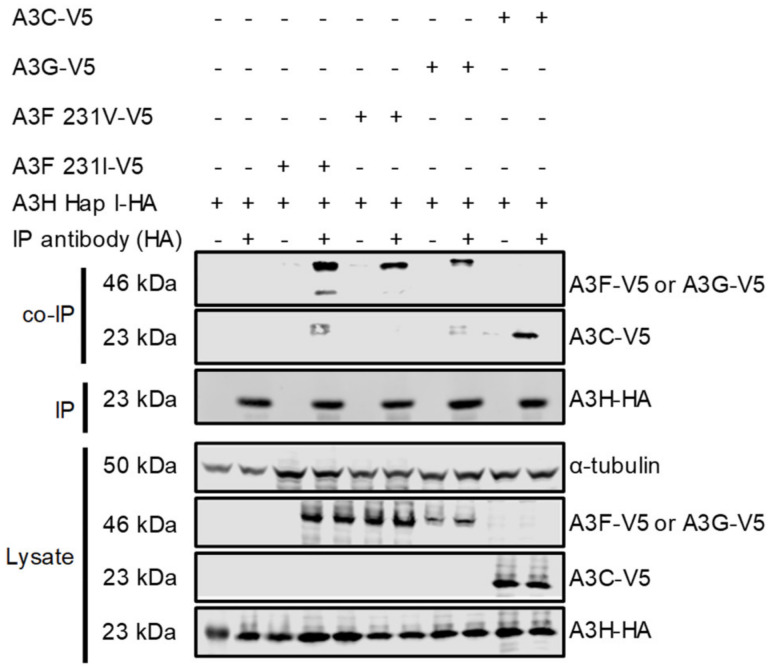
Multiple A3s interact with A3H Hap I. The immunoprecipitation from cell lysates used anti-HA antibody. Co-IP of A3H Hap I-HA with A3F 231I-V5, A3F 231V-V5, A3G-V5 or A3C-V5 was detected through the V5-tag. The presence of HA-tagged A3H Hap I in the co-IP samples is shown. The experimental and mock samples are prepared from the same lysate. The lysate blot demonstrates the cellular expression of A3H Hap I-HA and V5-tag A3s. The α-tubulin was used as a loading control.

Although A3H Hap I is less stable in cells than other A3H haplotypes and other A3s, it is one of the most common A3H alleles in humans, can restrict HIV up to two-fold and is resistant to Vif-mediated degradation [33,47]. To determine if A3H Hap I can interact with other A3s, a co-IP was conducted with the following A3s: A3F, A3G, and A3C. For A3F, we used two polymorphic variants that occur in humans most often as heterozygous alleles of A3F 108A/231V (referred to as 231V) and A3F 108S/231I (referred to as 231I) [37]. The A3F 231V is more stable in cells than the A3F 231I [37]. We co-transfected equal amounts of HA-tagged A3H Hap I with V5-tagged A3F (231V or 231I), A3G, or A3C in HEK 293T cells. The co-immunoprecipitation was done in the presence of RNase A to disrupt any RNA-mediated interactions. All the V5-tagged A3s immunoprecipitated with A3H Hap I and indicated that protein-protein interactions occurred between A3H Hap I and A3F 231V, A3F 231I, A3G and A3C and were not mediated by RNA (Figure 1). Although the presence of an intermediate protein bridging two APOBEC3 proteins cannot be ruled out, it is unlikely based on the similar amino acid sequences of A3 proteins and their tendency to oligomerize, and biochemical data with purified A3F and A3G showed that they interact through a direct protein-protein manner [44].

### 3.2. A3F Gains Partial Protection against Vif-Mediated Ubiquitination at the Expense of A3H Hap I

To determine if stable A3 enzymes could alter A3H Hap I stability in cells, we co-expressed HA-tagged A3H Hap I with V5-tagged A3F, A3G, or A3C in HIV (−Vif and +Vif) producer cells. To guarantee the co-expression of A3H Hap I and the other A3 per transfected cell, we used the pVIVO2 vector, which has two MCS enabling co-expression of two A3 enzymes from the same plasmid (see Material and Methods). We used increasing amounts of pVIVO2 vector (25 ng, 50 ng, and 100 ng) that expressed A3H Hap I-HA alone, A3H Hap I-HA with other V5-tagged A3s, or V5-tagged A3s alone. The different plasmid amounts represent low, middle and high expression levels of A3 enzymes, since it is difficult to predict the amount of steady state enzyme in CD4+ T cells as this is expected to differ between people [11].

**Figure 2:**
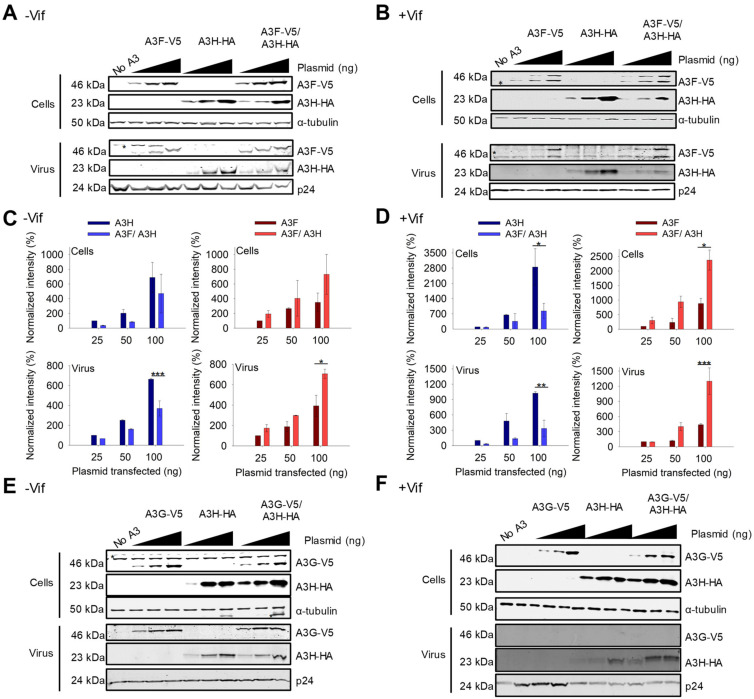
A3F gains partial resistance to Vif-mediated degradation when co-expressed with A3H Hap I. (**A**,**B**) Immunoblots of demonstrating protein levels of A3F-V5, A3H Hap I-HA and co-expressed A3F-V5/A3H Hap I-HA in cells and virions. An increasing amount of transfected A3 plasmid was used for each transfection condition (25 ng, 50 ng, and 100 ng) for (**A**) −Vif and (**B**) +Vif conditions. The non-specific bands are denoted by an asterisk. The α-tubulin and p24 were used as loading controls for cell lysates and virions, respectively. One representative blot is shown. (**C**,**D**) Analysis of immunoblots shown in A-B and their replicates. The immunoblots were conducted in three independent experiments and analyzed to determine the band intensity of the A3 with each normalized to the loading control in the same sample (α-tubulin for cell lysates and p24 for viral lysates). The intensity of HA tagged A3H Hap I alone (dark blue) and in co-expression with A3F (light blue) in the absence and presence of Vif was analyzed. The intensity of V5-tagged A3F alone (dark red) and in co-expression with A3H Hap I (light red) in absence and presence of Vif was analyzed. Values are normalized to the 25 ng transfection condition for A3H or A3F alone. Error bars represent the standard deviation of the mean from three independent experiments. Designations for significant difference between values of the same transfection condition are shown as: *p* ≤ 0.001 (***), *p* ≤ 0.01(**), or *p* ≤ 0.05 (*). (**E**,**F**) Immunoblots as shown in A-B, but detecting A3G-V5, A3H Hap I-HA and co-expressed A3G-V5/A3H Hap I-HA in cells and virions.

For A3F and A3H Hap I, we observed that the steady state expression levels of the proteins changed when they were co-expressed as compared to their individual expression. Similar results were found with A3F 231V (Figure 2A,B) and A3F 231I (Figure S1A,B). In −Vif conditions, across all transfection conditions, A3F encapsidation and protein levels were improved ~two-fold when co-expressed with A3H Hap I, while A3H Hap I experienced a ~two-fold decrease in the steady state expression level when co-expressed with A3F 231V compared to A3H Hap I alone (Figure 2A,C). In presence of Vif, the steady state expression level of A3F was less than in the absence of Vif, but A3H Hap I expression was similar in agreement with previous data showing A3H Hap I is resistant to Vif (Figure 2A–D) [48]. However, when co-expressed the results were similar to −Vif conditions and across all transfection conditions A3H Hap I protein levels in cells as well as encapsidation levels in virions were reduced ~six-fold when A3H Hap I was co-expressed with A3F compared to when expressed alone (Figure 2B,D). In contrast, the A3F protein levels in cells and virions in presence of A3H Hap I were consistently increased ~five-fold compared to A3F alone (Figure 2B,D). Since the steady state expression level of A3H Hap I when co-expressed with A3F is less in +Vif conditions than −Vif conditions, we concluded that A3H Hap I in presence of A3F becomes more sensitive to Vif-mediated degradation and more unstable in cells, while A3F gains partial protection from Vif-mediated degradation.

We investigated if other A3s that interacted with A3H Hap I can also affect A3H Hap I steady state protein levels and virion encapsidation. In absence or presence of Vif, A3G steady state protein levels in cells and encapsidation levels in virions were the same when expressed alone or with A3H Hap I (Figure 2E,F). This is especially evident in the presence of Vif where A3G is highly sensitive to Vif-mediated degradation, resulting in nearly undetectable levels of A3G in virions (Figure 2F). Similarly, A3H Hap I steady state protein levels in cells and encapsidation levels in virions did not change when co-expressed with A3G (Figure 2E,F). Similar results were observed with A3C (Figure S2A,B). Altogether, it appears that the A3-induced increase in A3H Hap I instability in cells is unique to A3F.

### 3.3. HIV Restriction after Co-Expression of A3F and A3H Hap I in the Absence and Presence of Vif

**Figure 3:**
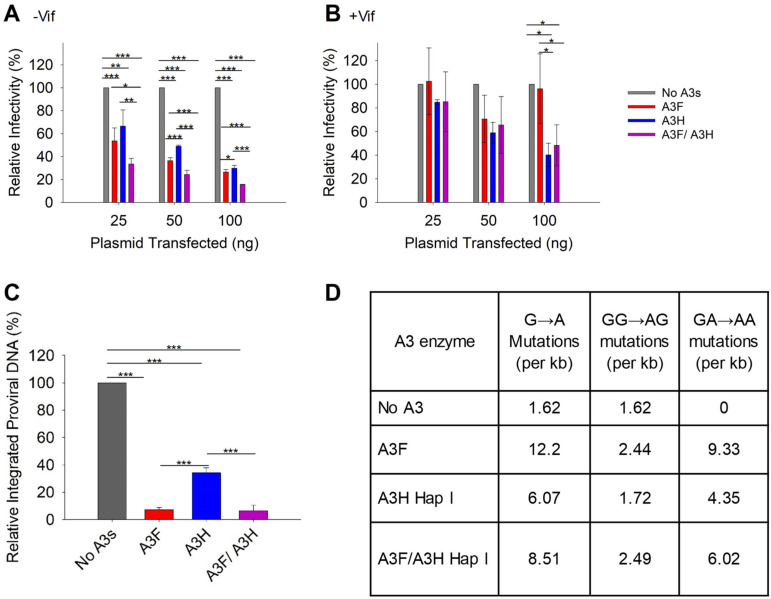
Restriction of HIV by A3F and A3H Hap I alone and when co-expressed. (**A**,**B**) Infectivity was measured via β-galactosidase in TZM-bl cells infected with HIV in the absence (**A**) −Vif and presence (**B**) +Vif when A3F and A3H Hap I were expressed alone or together in virus producer cells. (**C**) Quantification of relative −Vif HIV pro-viral DNA integrated into the host genome using qPCR when 100 ng of A3s were transfected into virus producing cells. The error bars represent the standard deviations of the mean from three independent experiments. Designations for significant difference between values of the same transfection condition are shown as: *p* ≤ 0.001 (***), *p* ≤ 0.01(**), or *p* ≤ 0.05 (*). (**D**) Analysis of A3-induced mutagenesis in −Vif HIV pro-viral DNA was carried out via PCR amplification of a 581 bp region of *pol* when 100 ng of A3s were transfected into virus producing cells. Clones were sequenced, aligned with Cluster Omega, and analyzed using Hypermut. The total base pairs sequenced for each condition were: No A3: 9877; A3F: 6972; A3H Hap I: 11,039; A3F/A3H Hap I: 10,440.

With the loss of A3H Hap I when expressed with A3F, we did not expect to see a synergistic effect of co-expressed A3F and A3H Hap I on infectivity. However, it was important to characterize if there were any effects on HIV infectivity, since A3H Hap I is a common haplotype across multiple populations [35]. To determine the effect of A3F and A3H Hap I on HIV infectivity, we first established the ability of increasing amounts of A3F and A3H Hap I to restrict HIV in the absence or presence of Vif when expressed alone. In absence of Vif, A3F and A3H Hap I restrict HIV similarly, with the HIV having 60 to 30% infectivity when 25, 50, or 100 ng of the A3 expression plasmid was transfected into the virus producing cell (Figure 3A). In the presence of Vif, the A3F does not consistently decrease viral infectivity even at the 100 ng transfection level (Figure 3B). However, A3H Hap I does show a dose dependent decrease in viral infectivity, in accordance with its previously reported resistance to Vif-mediated degradation (Figure 3B) [48]. When A3F and A3H Hap I are co-expressed, it results in an ~two-fold increase in restriction in the absence of Vif for all transfection conditions (Figure 3A). However, despite A3H Hap I being at least partially resistant to Vif, in the presence of Vif this two-fold increased ability to restrict HIV is not observed (Figure 3B). Rather, the infectivity is not significantly different than A3H Hap I alone. These data are consistent with the immunoblotting data (Figure 2A–D). For −Vif conditions, an increase in restriction activity by A3F in the presence of A3H Hap I correlates with enhanced A3F encapsidation. For +Vif conditions, a partial resistance to Vif is observed when A3F and A3H Hap I are co-expressed, which correlates with enhanced A3F, but not A3H Hap I encapsidation (Figure 2A,B and Figure 3A,B). Similar results were found with A3F 231I (Figure S1C,D). Consistent with immunoblotting data, no effects on infectivity were observed when A3H Hap I was co-expressed with A3C (Figure S2C) or A3G (Figure S3).

A3 enzymes are known to restrict HIV by inducing mutagenesis or degradation of the pro-viral genome. Degradation of the pro-viral genome results from host DNA repair enzyme processing of uracils and results in less pro-viral DNA integration [7]. Deamination-independent effects, such as blocking reverse transcription can also result in less pro-viral DNA integration [7,17]. To determine the main restriction mechanism we quantified pro-viral DNA integration and mutations. The −Vif HIV pro-viral DNA integrated into the host genome in absence and presence of 100 ng transfected A3s was determined by qPCR. In presence of A3F, A3H Hap I, and A3F/A3H Hap I less pro-viral DNA was integrated in comparison to absence of A3s (Figure 3C). However, the decrease of pro-viral integration was similar for A3F alone and co-expressed A3F/A3H Hap I and approximately four-fold more than A3H Hap I alone (Figure 3C). Similar results were found with A3F 231I and A3H Hap I (Figure S1E). To determine the mutations that the enzymes induced, we PCR amplified and sequenced a 581 bp region of the *pol* gene from −Vif HIV pro-viral DNA (see Materials and Methods). The A3F recognition motif is 5’TTC/TC and for A3H is 5’CTC/TC although they also deaminate 5’CC motifs at a lower level (Figure 3D) [36,49]. We wondered if the co-expression conditions would have approximately two-fold higher levels of mutations. The comparison of A3F, A3H Hap I, and A3F/A3H Hap I induced mutation rates per kb is shown as total G to A mutations, GG to AG mutations (resulting from deaminations at CC motifs) and GA to AA mutations (resulting from deaminations at TC motifs). The data shows that although the mutation rate is increased when A3F and A3H Hap I co-encapsidated compared to A3H Hap I alone, it is roughly equal to the mutation rate of A3F alone, consistent with less A3H being present under these conditions (Figure 2A and Figure 3D). Altogether the results show that, in the absence of Vif, the enhanced encapsidation of A3F does not increase the mutation rate or decrease pro-viral DNA integration more than A3F alone. This may be due to the less efficient processivity of A3F on ssDNA [44].

### 3.4. A3F Increases the Proteasomal Degradation of A3H Hap I

**Figure 4:**
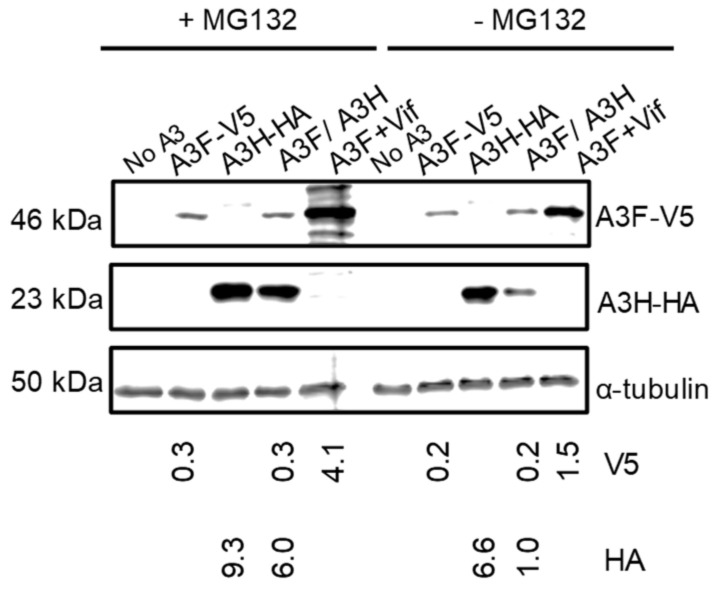
A3F promotes the proteasomal degradation of A3H Hap I. A) A3F and A3H Hap I were transfected into 239T cells alone or co-expressed. Twenty-four hours after the transfection, MG132 (+MG132) was added to block proteasomal degradation or DMSO (−MG132) was added as a mock. As a positive control for the effectiveness of the MG132 treatment, A3F was co-expressed with Vif. The MG132 rescued the steady state levels of A3F in the presence of Vif. The α-tubulin was used as a loading control.

Since A3H Hap I is known to be more rapidly ubiquitinated and degraded in cells than A3H Hap II, we hypothesized that A3F may be promoting A3H Hap I proteasomal degradation [32]. To test this, we used the proteasome inhibitor MG132 to determine if in the presence of A3F, MG132 could recover steady state A3H Hap I protein levels to that of A3H Hap I alone. We transfected equal amounts of A3 plasmids in the absence of HIV and treated them with MG132. We also transfected A3F in the presence of Vif, since Vif induces proteasomal degradation of A3F, and used this as a positive control for MG132 blocking proteasomal degradation (Figure 4). The MG132 did rescue A3F steady state expression levels in the presence of Vif (Figure 4). The results also showed that, in the presence of MG132, the amount of A3H Hap I in the presence of A3F was six-fold more than in the absence of MG132 (Figure 4). We did find an approximately 1.5-fold recovery of A3H Hap I alone in the presence of MG132, consistent with its enhanced ubiquitination in cells (Figure 4) [32]. A3F protein levels in both alone and co-expressed with A3H Hap I were similar in the presence and absence of MG132 (Figure 4). Thus, A3F leads to increased A3H Hap I proteasomal degradation.

## 4. Discussion

All A3 enzymes, except A3A, are co-expressed in CD4^+^ T cells and all the A3s that localize to the cytoplasm can co-encapsidate into virions [10,11,50]. However, our understanding of how these A3 enzymes influence each other’s activities is limited [24,25,50]. Here we examined A3H Hap I. The A3H Hap I frequency in humans is approximately 50% with some ethnicities reaching 90% allele frequency [33,35]. A3H Hap I is able to restrict HIV, but less than A3H Hap II, A3F, or A3G [31,32,33,35]. A3H Hap I does not restrict HIV well since it is targeted for ubiquitination and degradation at a higher rate than A3H Hap II and other A3 enzymes [32]. We hypothesized that A3H hetero-oligomerizing with more stable A3 enzymes may enhance A3H Hap I activity. Chimeras of A3H Hap II or A3C with A3H Hap I have shown increased stability and ability to restrict HIV, but they are not naturally occurring [32,51]. We found that A3H Hap I has the ability to hetero-oligomerize with A3C, A3F and A3G, but the interactions of A3C and A3G had no effect on A3H Hap I steady state levels and, surprisingly, the interaction with A3F accelerated proteasomal degradation of A3H Hap I. This at the same time led to increased A3F encapsidation in the absence and presence of Vif. These data demonstrate that A3 enzymes may co-regulate each other at the protein level. This is important for A3 enzymes that have been implicated in somatic mutagenesis and cancer, such as A3H Hap I [52].

A3H Hap I can interact with multiple A3 enzymes. The ability of A3 enzymes to hetero-oligomerize and influence HIV infectivity was first found with A3G and A3F [24]. This study shows that hetero-oligomerization appears to be a more general phenomenon, likely owing to similar amino acid sequences for all the A3 enzymes. However, not all the interactions were functional or functional in the same manner. The A3F and A3G interaction resulted in enhanced HIV restriction and a protection of A3F from Vif-mediated degradation [25]. However, we observed that A3F accelerated A3H Hap I degradation. Nonetheless, A3F steady state levels in cells and virions increased at the expense of A3H Hap I. This resulted in two-fold more restriction of HIV in the absence of Vif and enabled A3F to restrict HIV up to two-fold in the presence of Vif. This interaction was functional, although not in the way that was expected. Perhaps different results were observed due to the different holoenzyme structure of A3H. A3H is an obligate dimer with a double-stranded RNA and the RNA influences enzyme activity and protein stability [26,27,28,29,30]. The mechanism by which A3F promotes A3H degradation in the proteasome is not known, but it is plausible that the A3F/A3H Hap I hetero-oligomer causes A3H to dissociate from the bound RNA, resulting in accelerated degradation. If this type of general mechanism is occurring then A3F may accelerate proteasomal degradation of other A3H haplotypes. Presumably, with other more stable haplotypes, such as A3H Hap II, the effect would be less since there would be higher steady state levels of A3H Hap II in cells. However, this mechanistic hypothesis does not explain why there is more A3F in cells and virion encapsidated. These results may suggest some competition between A3s for virion encapsidation.

Co-regulation between A3s has been identified previously. When A3H Hap I is made into a chimeric two domain enzyme by linking it to A3C through a peptide, the HIV restriction efficiency of A3H Hap I and A3C increased by two orders of magnitude [51]. While artificially linking two enzymes with low restriction ability increased the activity against HIV, the natural hetero-oligomerization of A3C and A3H Hap I had no effect. However, a natural regulatory mechanism has been identified with other A3s. A3D has been found to suppress entry of A3F and A3G into Hepatitis B virions, which decreases their restriction ability [53]. A3A and A3B can be expressed in the same cell in some cancers, such as breast tumor cells [54]. A3A, A3B, and A3H Hap I are known to cause somatic mutations in tumor cells [55,56,57]. It was found that deletion of A3B resulted in higher steady state levels of A3A and increased A3A-induced mutagenesis in cancer cells, but the mechanism of co-regulation was not known [54]. A3H Hap I is less active than A3A and A3B, but does contribute to somatic mutations found in breast, lung and possibly other cancers [52]. Although A3F has not been found to be co-expressed with A3H Hap I in breast or lung tumors, the potential loss of A3H Hap I in CD4+ T cells due to A3F may decrease the chance of off target somatic mutations.

These data demonstrate that some A3-A3 interactions may not be synergistic or even additive. As a result, the restriction efficiency of A3 enzymes needs to be taken as a whole rather than individually since they can influence each other’s deamination activity and cellular stability. This is especially important in the presence of Vif. That there are high levels of A3-induced mutations in HIV pro-viral genomes isolated as early as 6 weeks after infection has suggested that either Vif is not entirely effective or A3 enzymes have mechanisms to resist Vif [22]. The data presented here demonstrate that co-expression of A3H Hap I and A3F can enable partial restriction of HIV by A3F in the presence of Vif, which may be part of the reason that A3 enzymes can induce mutations in pro-viral DNA early in infection. Other contributions can be made by A3F and A3G hetero-oligomers and possibly other A3 interactions that are yet to be discovered [24,25].

## Data Availability

Data is contained within the article or Supplementary Materials.

## Supplementary Materials

The following supporting information can be downloaded at: https://www.mdpi.com/article/10.3390/v15020463/s1, Figure S1: A3F 231I gains partial resistance to Vif-mediated degradation when co-expressed with A3H Hap I; Figure S2: Restriction of HIV by A3C and A3H Hap I alone and when co-expressed; Figure S3: Restriction of HIV by A3G and A3H Hap I alone and when co-expressed.
