# Supplementary material for: Stability of APOBEC3F in the Presence of the APOBEC3 Antagonist HIV-1 Vif Increases at the Expense of Co-Expressed APOBEC3H Haplotype I
Source: Viruses. 2023 Feb 7;15(2):463. doi: 10.3390/v15020463 (PMC9960753; doi:10.3390/v15020463)
Supplement: Supplementary file 1 [file viruses-15-00463-s001.zip › viruses-2159473-supplementary.pdf]

Supplementary Data

Stability of APOBEC3F in the presence of the APOBEC3 an-tagonist HIV-1 Vif increases at the expense of co-expressed APOBEC3H Haplotype I

Maria Yousefi[1], Arun Kumar Annan Sudarsan[1,2], Amit Gaba[1], and Linda Chelico[1]*

[1]University of Saskatchewan, College of Medicine, Department of Biochemistry, Microbiology, and Immunology, Saskatoon, Saskatchewan, Canada, S7N 5E5

[2]Current address: Centre for Commercialization of Regenerative Medicine (CCRM), 661 University Ave #1002, Toronto, ON M5G 1M1

*Corresponding author email: linda.chelico@usask.ca

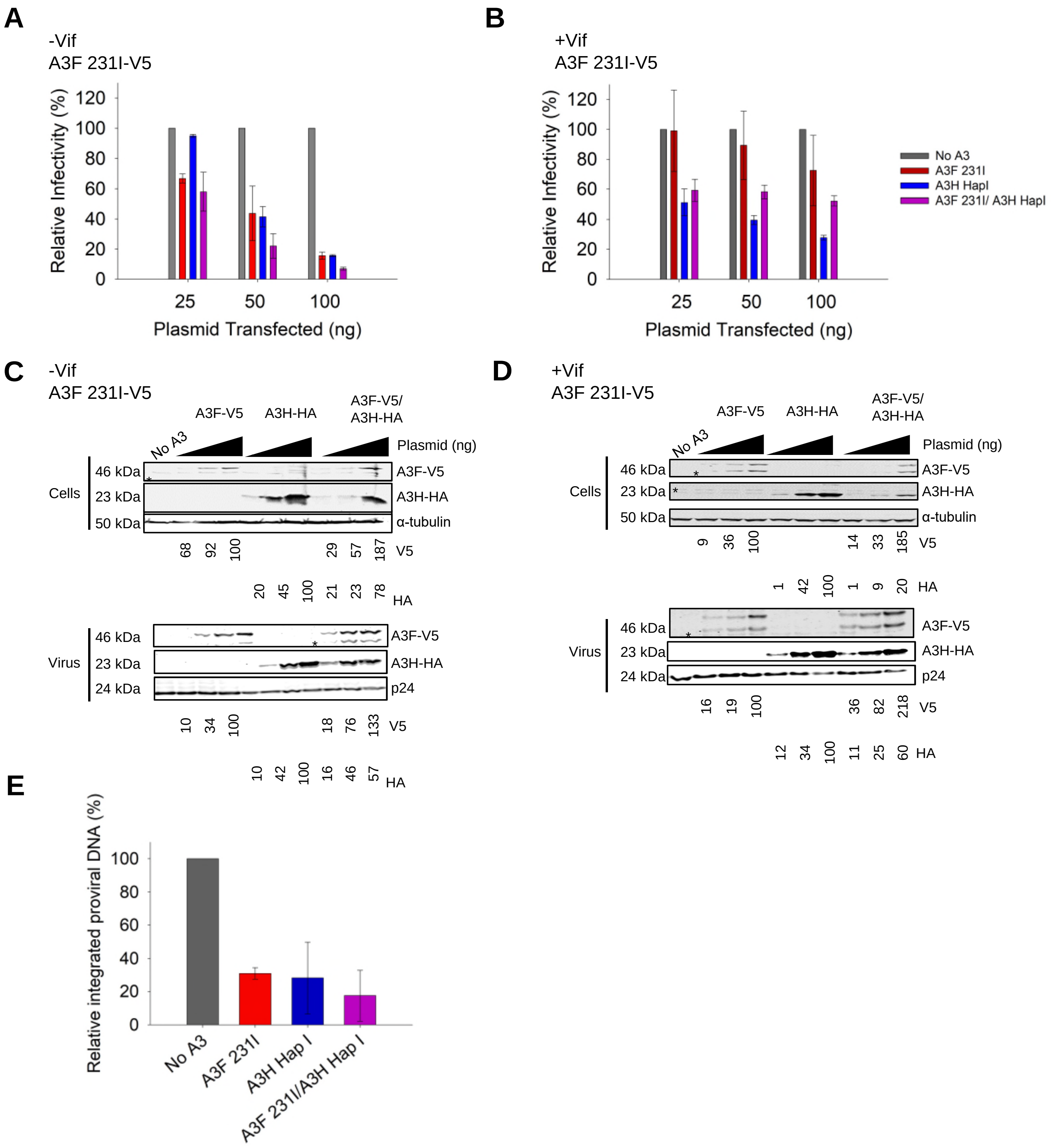

Figure S1. A3F 231I gains partial resistance to Vif-mediated degradation when co-expressed with A3H Hap I. A-B) Infectivity was measured in the absence (A) -Vif and presence (B) +Vif when A3F 231I, A3H Hap I were expressed alone or together in virus producer cells. C-D) Immunoblots of demonstrating protein levels of A3F 231I-V5, A3H Hap I-HA and co-expressed A3F 231I-V5/ A3H Hap I-HA in cells and virions. An increasing amount of transfected A3 plasmids was used for each transfection condition (25 ng, 50 ng, and 100 ng) for C) -Vif and D) +Vif conditions. The non-specific bands are illustrated by an asterisk. The α-tubulin and p24 were used as loading controls for cell lysates and virions, respectively. The band intensity of the A3 was calculated by normalizing each A3 (V5 or HA) band to the loading control in the same sample (α-tubulin for cell lysates and p24 for viral lysates). For comparison, the 100 ng condition for each A3 was set to 100%. One representative blot is shown. E) Quantification of relative proviral DNA integrated into the host genome using qPCR when 100 ng of A3s were transfected into virus producing cells. The error bars represent the standard deviations of the mean from three independent experiments.

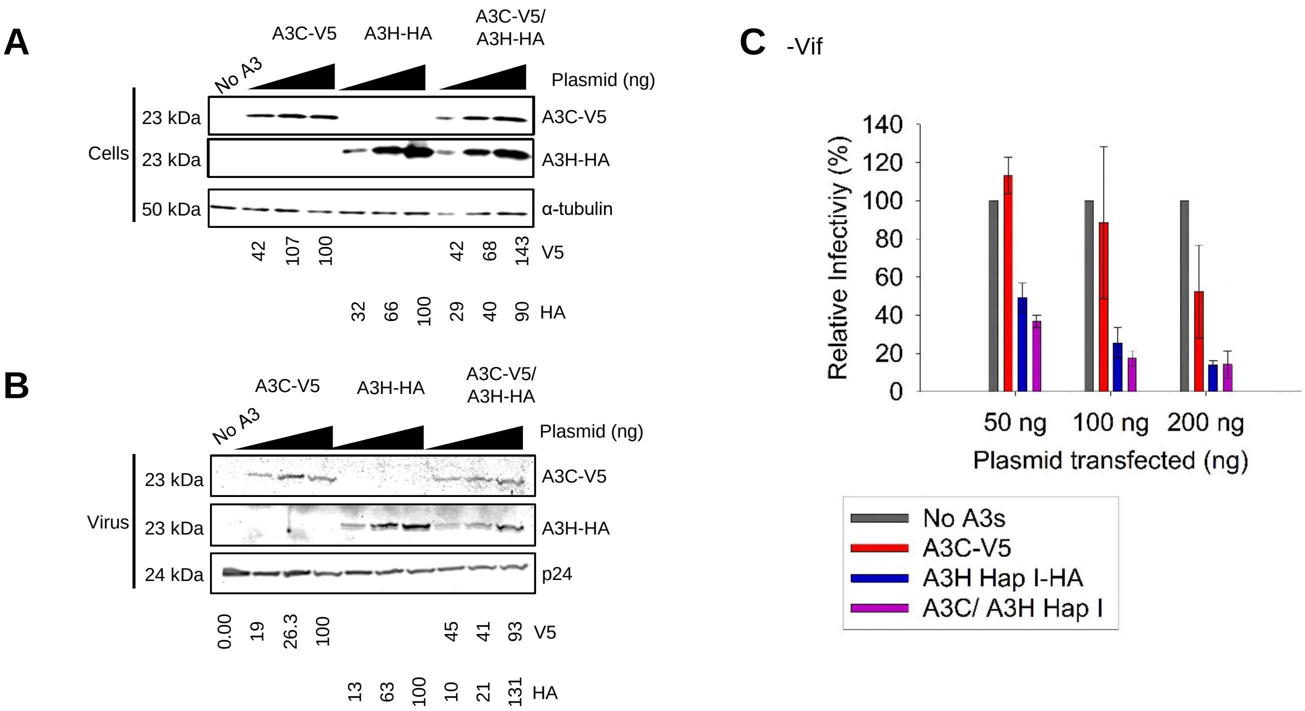

Figure S2. Restriction of HIV by A3C and A3H Hap I alone and when co-expressed. A-B) Immunoblots of demonstrating protein levels of A3C, A3H Hap I-HA and co-expressed A3C/ A3H Hap I-HA in cells and virions. An increasing amount of transfected A3 plasmids was used for each transfection condition (25 ng, 50 ng, and 100 ng). The non-specific bands are illustrated by asterisk. The α-tubulin and p24 were used as loading controls for cell lysates and virions, respectively. The band intensity of the A3 was calculated by normalizing each A3 (V5 or HA) band to the loading control in the same sample (α-tubulin for cell lysates and p24 for viral lysates). For comparison the 100 ng condition for each A3 was set to 100%. One representative blot is shown. C) Infectivity was measured via β-galactosidase in TZM-bl cells infected with HIV in the absence of Vif (-Vif). The error bars represent the standard deviations of the mean from three independent experiments.

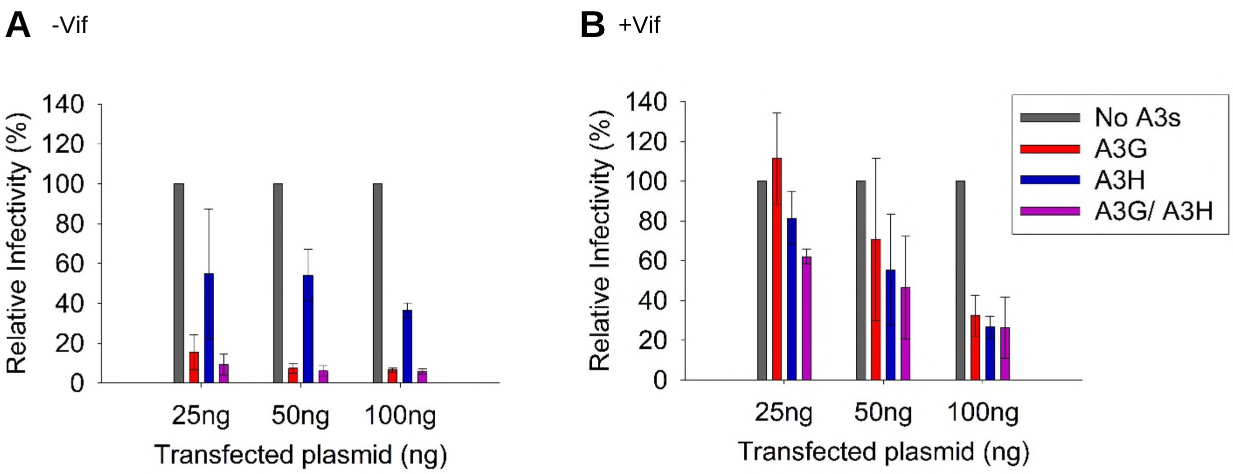

Figure S3. Restriction of HIV by A3G and A3H Hap I alone and when co-expressed. A-B) Infectivity was measured via β-galactosidase in TZM-bl cells infected with HIV in the absence (A) -Vif and presence (B) +Vif when A3G, A3H Hap I were expressed alone or together in virus producer cells. The error bars represent the standard deviations of the mean from three independent experiments.
